# On the Complementarity of the Harmonic Oscillator Model and the Classical Wigner–Kirkwood Corrected Partition Functions of Diatomic Molecules

**DOI:** 10.3390/e22080853

**Published:** 2020-07-31

**Authors:** Marcin Buchowiecki

**Affiliations:** Insitute of Physics, University of Szczecin, Wielkopolska 15 St, 70-451 Szczecin, Poland; marcin.buchowiecki@usz.edu.pl

**Keywords:** vibrational partition function, rovibrational partition function, Wigner–Kirkwood expansion

## Abstract

The vibrational and rovibrational partition functions of diatomic molecules are considered in the regime of intermediate temperatures. The low temperatures are those at which the harmonic oscillator approximation is appropriate, and the high temperatures are those at which classical partition function (with Wigner–Kirkwood correction) is applicable. The complementarity of the harmonic oscillator and classical integration over the phase space approaches is investigated for the CO and H${}_{2}^{+}$ molecules showing that those two approaches are complementary in the sense that they smoothly overlap.

## 1. Introduction

In certain applications such as the investigation of the equation of state, a very large temperature range is considered. In some research [1,2], even though the extremely high and extremely low temperatures are considered, the harmonic oscillator is used for the description of vibrations (with a cut-off to describe dissociation); moreover, the effect of rovibrational coupling is not mentioned despite the fact that it exerts a profound and nonlinear effect on the partition function at high temperatures [3].

Some studies neglect dissociation and consider only bound states at high temperatures in the rovibrational partition function because of the unrealistic (harmonic) potential energy curve [4]. Some other studies take into account anharmonicity and rovibrational coupling [5,6], but still do not consider unbound states [7], which are easily included in the partition function in the classical approach [8,9,10,11,12,13]; thermodynamical data assume the ideal-gas approach, and the unbound states’ effect is not negligible at high temperatures common in plasma science and hypersonic flows.

In principle, none of the above simplifications of the partition function for diatomic molecules are needed, but a high quality partition function can be obtained from the quantum corrected classical approach. The condition for that statement is the availability of exact potential energy curves, which in practice are not always available, especially for excited electronic states of molecules.

The Wigner–Kirkwood expansion [14,15] some years ago was claimed to be impractical to use for the vibrational partition function [16]. I will show, despite the fact that the low temperature limit is indeed incorrect, that the three terms of expansion can be used effectively (and are complementary to the harmonic oscillator model) for both vibrational and rovibrational partition functions.

Arbitrary high temperatures were considered in the already mentioned publications [11,13]. At low temperatures, the harmonic oscillator model (HO, for the vibrational partition function) and the rigid rotor harmonic oscillator model (RRHO, for the rovibrational partition function) can be used unless the temperature is not very low and the anharmonicity of zero point energy is not significant. In this study, this effect is very small, and the multiplicative correction factor $e^{-\beta E_{0}}e^{\beta \omega /2}$ (to remove harmonic zero point energy ω/2 and replace it with the exact zero point energy $E_{0}$ in harmonic approximation; atomic units are utilized) is not used. In the case of the CO molecule, this correction is around 0.5% at temperatures of 800–1000 K and almost 2% at 100 K. Note that higher vibrational states are less important with decreasing temperature so that the above described correction works in a wide range of temperatures.

The aim of this paper is to show that the results of the harmonic oscillator method and classical Wigner–Kirkwood corrected method give almost the same results in a certain temperature range. This overlap means that those methods are complementary and together are able to give ideal-gas partition functions in a very broad range of temperatures.

## 2. Methods

The partition function of CO was calculated on the Liu potential energy curve [17]:

(1) $$\begin{array}{ccc} V_{Liu} & = & -D_{e}[1+a_{1}\left(r-r_{e}\right)+a_{2}{\left(r-r_{e}\right)}^{2}\\ & + & a_{3}{\left(r-r_{e}\right)}^{3}]exp\left[-a_{1}\left(r-r_{e}\right)\right]+D_{e}, \end{array}$$

where $D_{e}=0.4113827a.u.$, $r_{e}=2.13955.a.u.$, $a_{1}=2.20355a.u.$, $a_{2}=0.962467.a.u.$, $a_{3}=0.408807a.u.$ (atomic units are used). The angular frequency for this PECis ω=0.00982934a.u., and the reduced mass for the ^12^C^16^O isotope is μ=12498.1a.u..

The potential energy curve for H${}_{2}^{+}$ according to [18] is:

(2) $$\begin{array}{ccc} V_{XG}\left(r\right) & = & 0.1026+\frac{\left(\exp\left(-4.5r\right)\left(1+\frac{1}{r}\right)+\exp\left(-1.05111r\right)\left(\frac{1}{r}-0.917034r\right)\right)}{\left(1+\exp\left(-r\right)\left(1+r+r^{2}/3\right)\right)}, \end{array}$$

with ω=0.0104506a.u, reduced mass μ=918.576.

The partition functions are calculated according to the ideal gas approach, which takes into account bound, metastable, and scattering states [7]. The vibrational partition function [13]:

(3) $$Q_{vib}^{\mathrm{HD},\mathrm{wk}}=\frac{1}{2\pi }\sqrt{\frac{2\pi \mu }{\beta }}\int_{0}^{\infty }\left[\exp\left(-\beta V\right)wk\left(r\right)-\exp\left(-\beta D_{e}\right)\right]dr,$$

and the rovibrational partition function [10,11] (the symmetry number 1/2 has to be included for homonuclear molecules):

(4) $$Q_{rovib}^{\mathrm{HD},\mathrm{wk}}=\frac{1}{2\sqrt{\pi }}{\left(\frac{2\mu }{\beta }\right)}^{3/2}\int_{0}^{\infty }\left[\exp\left(-\beta V\right)wk\left(r\right)-\exp\left(-\beta D_{e}\right)\right]r^{2}dr,$$

where $\beta =1/(k_{B}T)$ is the inverse temperature, *V* the potential energy function of the molecule under consideration, and $D_{e}$ the depth of the potential energy curve. In both formulas, exp(−βV) was multiplied by wk(r), which is the Wigner–Kirkwood quantum correction; for the vibrational partition function (one-dimensional case), it reads [19]:

(5) $$\begin{array}{ccc} wk_{vib}\left(r\right) & = & 1-\frac{\beta^{3}}{24\mu }{\left(V^{\prime }\right)}^{2}+\frac{\beta^{4}}{5760\mu^{2}}\left[\beta^{2}{\left(V^{\prime }\right)}^{4}-8\beta {\left(V^{\prime }\right)}^{2}V^{\prime \prime }+12{\left(V^{\prime \prime }\right)}^{2}\right]+\frac{1}{362880}{\left(\frac{\beta }{2\mu }\right)}^{3}[3\beta^{6}{\left(V^{\prime }\right)}^{6}+\\ & + & 12\beta^{4}{\left(V^{\prime }\right)}^{2}{\left(V^{\prime \prime }\right)}^{2}-216\beta^{2}{\left(V^{\prime \prime \prime }\right)}^{2}-50\beta^{5}{\left(V^{\prime }\right)}^{4}V^{\prime \prime }+480\beta^{3}V^{\prime }V^{\prime \prime }V^{\prime \prime \prime }], \end{array}$$

and for the rovibrational partition function, the correcting factor is [19,20]:

(6) $$\begin{array}{ccc} wk_{rovib}\left(r\right) & = & 1-\frac{\beta^{3}}{24\mu }{\left(V^{\prime }\right)}^{2}+\frac{\beta^{4}}{5760\mu^{2}}\left[\beta^{2}{\left(V^{\prime }\right)}^{4}-8\beta {\left(V^{\prime }\right)}^{2}r^{-2}d\left[r^{2}\left(V^{\prime }\right)\right]/dr+12{\left(r^{-2}d\left[r^{2}\left(V^{\prime }\right)\right]/dr\right)}^{2}\right]+\\ & + & \frac{\beta^{5}}{16\mu^{3}}[\frac{V^{\prime \prime \prime }}{840}+\frac{{\left(V^{\prime \prime }\right)}^{2}}{140r^{2}}+\frac{\beta }{726}{\left(V^{\prime \prime }\right)}^{3}+\frac{\beta }{180}\frac{V^{\prime }{\left(V^{\prime \prime }\right)}^{2}}{r}+\frac{\beta }{945}\frac{{\left(V^{\prime }\right)}^{3}}{r^{3}}-\\ & - & \frac{\beta^{2}}{720}{\left(V^{\prime }\right)}^{2}{\left(V^{\prime \prime }\right)}^{2}-\frac{\beta^{2}}{6480}\frac{{\left(V^{\prime }\right)}^{4}}{r^{2}}-\frac{\beta^{3}}{2160}\frac{{\left(V^{\prime }\right)}^{5}}{r}+\frac{\beta^{4}}{25920}{\left(V^{\prime }\right)}^{6}]. \end{array}$$

The correcting expression wk(r) is a series in which consecutive terms can be added for quantum correction of the classical partition function: wk0(r)=1 (classical case), where wk1(r) means using one correcting term, wk2(r) two correcting terms, and wk3(r) three correcting terms.

Finally, even if Wigner–Kirkwood correcting terms are used, the whole calculation amounts to a single one-dimensional numerical integration. The energy level based calculation, being preferable at lower temperatures, is more involved at the higher temperatures, and the question of the cut-off and quality of the high energy levels is crucial [21].

## 3. Results

### 3.1. Vibrational Partition Function of CO

**Figure 1:**
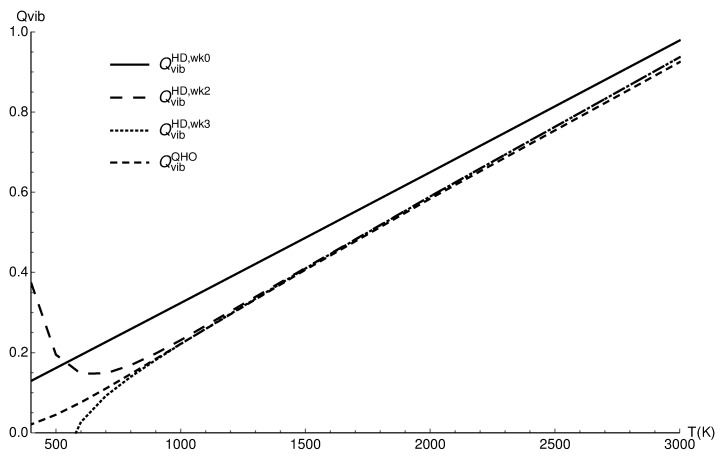
The vibrational partition functions of CO in the quantum harmonic approximation ($Q_{vib}^{\mathrm{QHO}}$), the classical values ($Q_{vib}^{\mathrm{HD},\mathrm{wk}0}$), the two term quantum Wigner–Kirkwood correction ($Q_{vib}^{\mathrm{HD},\mathrm{wk}2}$), and the three term correction ($Q_{vib}^{\mathrm{HD},\mathrm{wk}3}$).

In Figure 1, the vibrational partition functions for carbon monoxide are compared: the quantum harmonic approximation ($Q_{vib}^{\mathrm{QHO}}$), the fully classical value ($Q_{vib}^{\mathrm{HD},\mathrm{wk}0}$; no correction), the two term quantum Wigner–Kirkwood correction ($Q_{vib}^{\mathrm{HD},\mathrm{wk}2}$), and finally, the three term correction ($Q_{vib}^{\mathrm{HD},\mathrm{wk}3}$). The plot shows that classical value deviates from all others (it would be adequate at much higher temperatures).

The quantum harmonic oscillator values and two and three term corrected values are very close between 900 K and 1400 K, confirming that in this range of temperatures, the anharmonicity is negligible. The unbound states are also negligible, but for the vibrational partition function, much higher temperatures are needed for this effect [13]. At the highest temperatures of the plot, it is seen that the harmonic oscillator values depart from two and three term corrected classical values (they are the same; at those temperatures, two term correction is sufficient), and that effect shows the growing role of anharmonicity. At the lowest temperatures of the plot, it is seen that three term corrected classical values are in better agreement with the harmonic approximation below 1000 K than the two term corrected values; two terms do not provide the sufficient inclusion of quantum effects at around 1000 K.

The overall conclusion is the complementarity of the quantum harmonic approximation and three term corrected classical results (for reference, those values are given in Table 1).

Table 2 compares the harmonic vibrational partition function ($Q_{vib}^{\mathrm{QHO}}$) with the harmonic value $e^{-\beta E_{0}}e^{\beta \omega /2}$ corrected for exact zero point energy ($Q_{vib}^{\mathrm{QHO},\mathrm{ZPE}}$) and the single term partition function based only on zero point energy ($e^{-\beta E_{0}}$); $E_{0}$ is the exact lowest vibrational level (zero point energy). At low temperatures only, the zero point energy is sufficient or almost sufficient for the vibrational partition function.

### 3.2. Rovibrational Partition Function of CO

**Figure 2:**
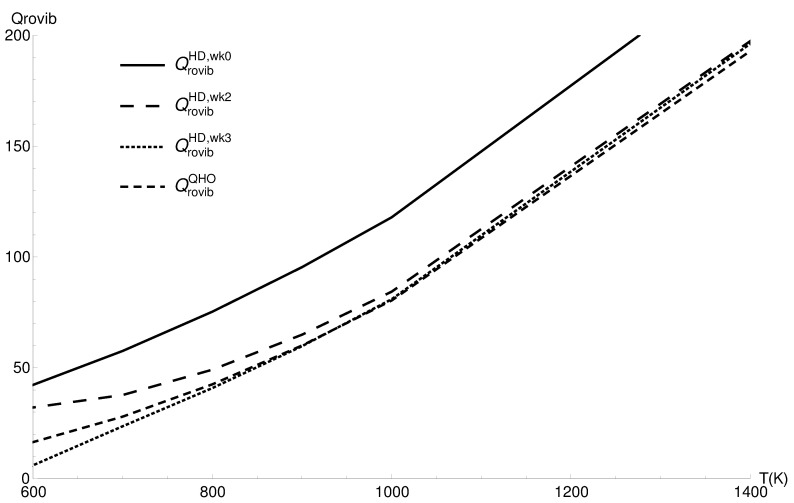
The rovibrational partition functions of CO in the quantum harmonic approximation ($Q_{rovib}^{\mathrm{QHO}}$), the classical values ($Q_{rovib}^{\mathrm{HD},\mathrm{wk}0}$), the two term quantum Wigner–Kirkwood correction ($Q_{rovib}^{\mathrm{HD},\mathrm{wk}2}$), and the three term correction ($Q_{rovib}^{\mathrm{HD},\mathrm{wk}3}$).

The vibrational partition function exhibits the same behavior as the vibrational partition function. In Figure 2, the region of the classical corrected and harmonic approximation’s best agreement is 900–1100 K; the same kind of complementarity is present.

In Table 3, the data of the rigid-rotor harmonic approximation and classical corrected method are given alongside the HITRANdatabase values. The agreement of rigid-rotor harmonic approximation with HITRAN values shows that anharmonicity and rovibrational coupling are very small effects below 1000 K for the CO molecule (classical corrected values do not include all the quantum effects below 1000 K, resulting in disagreement with the other values).

### 3.3. Vibrational and Rovibrational Partition Functions of H${}_{2}^{+}$

To confirm that the complementarity of harmonic and corrected classical values is not a random effect in the case of the carbon monoxide molecule, another molecule was also investigated. The molecular hydrogen ion H${}_{2}^{+}$ was chosen because it consists of hydrogen atoms and its bond is weak; all possible effects such as the quantum nature of the molecule, anharmonicity, and rovibrational coupling are more pronounced than in carbon monoxide.

**Figure 3:**
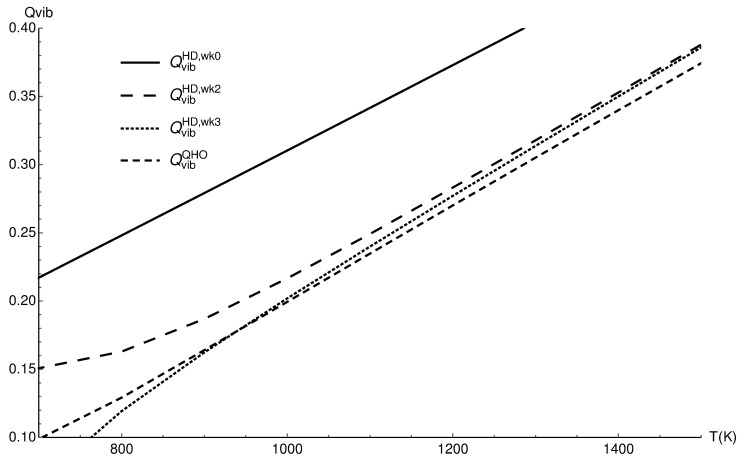
The vibrational partition functions of H${}_{2}^{+}$ in the quantum harmonic approximation ($Q_{vib}^{\mathrm{QHO}}$), the classical values ($Q_{vib}^{\mathrm{HD},\mathrm{wk}0}$), the two term quantum Wigner–Kirkwood correction ($Q_{vib}^{\mathrm{HD},\mathrm{wk}2}$), and the three term correction ($Q_{vib}^{\mathrm{HD},\mathrm{wk}3}$).

Figure 3 for the vibrational partition function shows that the complementarity is less pronounced, as could be expected: the regions of the harmonic approximation and three term corrected classical value overlap only in a very narrow range of temperatures.

**Figure 4:**
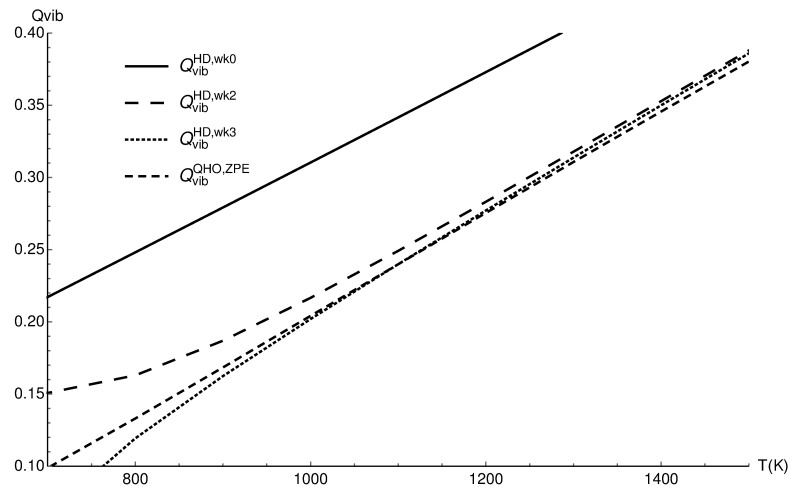
The vibrational partition functions of H${}_{2}^{+}$ in the quantum harmonic approximation with zero point energy correction ($Q_{vib}^{\mathrm{QHO},\mathrm{ZPE}}$), the classical values ($Q_{vib}^{\mathrm{HD},\mathrm{wk}0}$), the two term quantum Wigner–Kirkwood correction ($Q_{vib}^{\mathrm{HD},\mathrm{wk}2}$), and the three term correction ($Q_{vib}^{\mathrm{HD},\mathrm{wk}3}$).

In the case of this anharmonic molecular ion, the correction of zero point energy is more important than it is for carbon monoxide. Figure 4 repeats Figure 3, but the harmonic approximation is corrected with the accurate zero point energy; this correction causes the increased agreement of the harmonic approximation and the corrected classical results.

**Figure 5:**
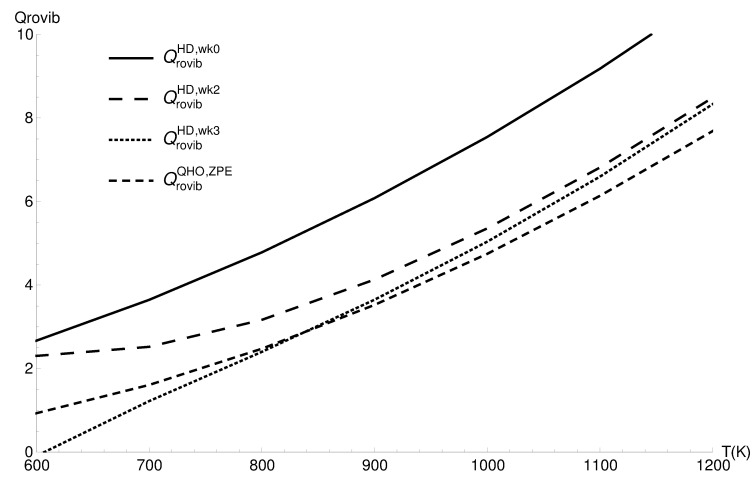
The rovibrational partition functions of H${}_{2}^{+}$ in the quantum rigid-rotor harmonic approximation with zero point energy correction ($Q_{rovib}^{\mathrm{QHO},\mathrm{ZPE}}$), the classical values ($Q_{rovib}^{\mathrm{HD},\mathrm{wk}0}$), the two term quantum Wigner–Kirkwood correction ($Q_{rovib}^{\mathrm{HD},\mathrm{wk}2}$), and the three term correction ($Q_{rovib}^{\mathrm{HD},\mathrm{wk}3}$).

In Figure 5, the rovibrational partition functions are compared, and the rigid-rotor harmonic approximation is zero point energy corrected. The complementarity region is very narrow. The uncorrected case of the rovibrational partition function (with slightly worse complementarity) is not shown.

It can be noted in general that for the H${}_{2}^{+}$ molecule, the two term quantum correction disagrees much more with the three term correction because of the greater quantum nature of that molecule in the same temperature range.

## 4. Conclusions

It is concluded that the partition functions of typical diatomic molecules exhibits the good complementarity of the harmonic oscillator approximation and Wigner–Kirkwood corrected classical method. Note especially that without the Wigner–Kirkwood correction, complementarity would not be achieved. In more loosely bounded diatomics (in particular molecular cations are bound more loosely than respective neutral molecules), the methods under consideration may be less complementary, and each temperature range needs more careful consideration when vibrational and rovibrational partition functions are calculated.

The Mathematica notebook with classical and Wigner–Kirkwood corrected vibrational and rovibrational partition functions of carbon monoxide in the 600–3000 K temperature range are deposited at the www.notebookarchive.org (accessed on 20 July 2020).

## Figures and Tables

**Table 1:** Vibrational partition function of carbon monoxide in harmonic approximation $Q_{vib}^{\mathrm{QHO}}$ and three term corrected classical values $Q_{vib}^{\mathrm{HD},\mathrm{WK}3}$.

T (K)	$Q_{\mathrm{vib}}^{\mathrm{QHO}}$	$Q_{\mathrm{vib}}^{\mathrm{HD},\mathrm{WK}3}$
600 K	$7.571\times 10^{-2}$	$2.647\times 10^{-2}$
700 K	$1.102\times 10^{-1}$	$9.198\times 10^{-2}$
800 K	$1.467\times 10^{-1}$	$1.396\times 10^{-1}$
900 K	$1.841\times 10^{-1}$	$1.816\times 10^{-1}$
1000 K	$2.218\times 10^{-1}$	$2.215\times 10^{-1}$
1500 K	$4.067\times 10^{-1}$	$4.098\times 10^{-1}$
2000 K	$5.840\times 10^{-1}$	$5.895\times 10^{-1}$
3000 K	$9.247\times 10^{-1}$	$9.369\times 10^{-1}$

**Table 2:** The carbon monoxide harmonic vibrational partition function ($Q_{vib}^{\mathrm{QHO}}$), the harmonic corrected for exact zero point energy ($Q_{vib}^{\mathrm{QHO},\mathrm{ZPE}}$), and the partition function based only on zero point energy ($e^{-\beta E_{0}}$).

T (K)	$Q_{\mathrm{vib}}^{\mathrm{QHO}}$	$Q_{\mathrm{vib}}^{\mathrm{QHO},\mathrm{ZPE}}$	$e^{-\beta E_{0}}$
200 K	$4.267\times 10^{-4}$	$4.382\times 10^{-4}$	$4.382\times 10^{-4}$
300 K	$5.667\times 10^{-3}$	$5.769\times 10^{-3}$	$5.769\times 10^{-3}$
400 K	$2.066\times 10^{-2}$	$2.094\times 10^{-2}$	$2.093\times 10^{-2}$
500 K	$4.497\times 10^{-2}$	$4.545\times 10^{-2}$	$4.536\times 10^{-2}$
600 K	$7.571\times 10^{-2}$	$7.639\times 10^{-2}$	$7.595\times 10^{-2}$
700 K	$1.102\times 10^{-1}$	$1.111\times 10^{-1}$	$1.098\times 10^{-1}$
800 K	$1.467\times 10^{-1}$	$1.477\times 10^{-1}$	$1.446\times 10^{-1}$
900 K	$1.841\times 10^{-1}$	$1.852\times 10^{-1}$	$1.793\times 10^{-1}$

**Table 3:** The carbon monoxide rovibrational partition function: rigid-rotor harmonic approximation $Q_{rovib}^{\mathrm{RRQHO}}$, three term corrected classical value $Q_{rovib}^{\mathrm{HD},\mathrm{WK}3}$, and values from the HITRAN database (HITRAN). For the sake of comparison with HITRAN, the zero point energy was removed; in parenthesis, the values with zero point energy are given.

T (K)	$Q_{\mathrm{rovib}}^{\mathrm{RRQHO}}$	$Q_{\mathrm{rovib}}^{\mathrm{HD},\mathrm{WK}3}$	HITRAN
600 K	$2.187\times 10^{2}$ ($1.646\times 10^{1}$)	$7.979\times 10^{1}$ ($6.001\times 10^{0}$)	$2.188\times 10^{2}$
700 K	$2.567\times 10^{2}$ ($2.796\times 10^{1}$)	$2.168\times 10^{2}$ ($2.361\times 10^{1}$)	$2.570\times 10^{2}$
800 K	$2.960\times 10^{2}$ ($4.254\times 10^{1}$)	$2.843\times 10^{2}$ ($4.086\times 10^{1}$)	$2.964\times 10^{2}$
900 K	$3.368\times 10^{2}$ ($6.005\times 10^{1}$)	$3.351\times 10^{2}$ ($5.975\times 10^{1}$)	$3.375\times 10^{2}$
1000 K	$3.794\times 10^{2}$ ($8.037\times 10^{1}$)	$3.821\times 10^{2}$ ($8.094\times 10^{1}$)	$3.803\times 10^{2}$
1500 K	$6.221\times 10^{2}$ ($2.211\times 10^{2}$)	$6.332\times 10^{2}$ ($2.250\times 10^{2}$)	$6.256\times 10^{2}$
2000 K	$9.195\times 10^{2}$ ($4.232\times 10^{2}$)	$9.397\times 10^{2}$ ($4.325\times 10^{2}$)	$9.283\times 10^{2}$
3000 K	$1.686\times 10^{3}$ ($1.005\times 10^{3}$)	$1.738\times 10^{3}$ ($1.036\times 10^{3}$)	$1.717\times 10^{3}$
